# A Systematic Scoping Review of Measures of the Quality of Health and Social Care for Adults in the Criminal Justice System: Learning for the Probation Service

**DOI:** 10.1177/02645505231221228

**Published:** 2024-01-23

**Keywords:** Quality indicators, standards, health, social care, criminal justice system, review

## Abstract

We conducted a systematic scoping review to identify quality indicators, guidelines or standards measuring the quality of health or social care for adult criminal justice populations that were relevant to probation practice. Seventeen papers were included, and measures were categorised by how they were developed, which national probation service health and social care strategy objectives they relate to, type of measures, and what is measured. We aimed to identify existing indicators that may support improvements in the health of people on probation and the care that they receive, and to share any insights around developing and introducing such indicators in practice.

## Background

Internationally, quality indicators are employed as part of quality improvement cycles in health and social care settings, including within the criminal justice system, to monitor and improve the quality of care that people receive. They can increase understanding of what works and where improvement efforts are needed and help to ensure that high-quality care is available for all.

Quality indicators may measure aspects of organisational structure (including human and material resources), processes involved in care giving (including referrals, and technical and interpersonal aspects of administering care), and outcomes (including changes to health status and satisfaction with care) (Donabedian, 1988).

Contact with the criminal justice system (CJS) provides an opportunity to identify health problems and to engage individuals that often do not access care except at crisis point and have poor past experiences of care. Improving the health of people in the CJS may produce benefits for individuals and for wider society through a ‘community dividend’ including supporting desistance from offending, reducing health inequalities and avoidable use of crisis care such as Accident and Emergency, and producing cost savings for health and criminal justice services (Revolving Doors Agency, 2017).

People on probation are expected to access health and social care in the same way as the wider population and may also engage with care as part of their sentence through community sentence treatment requirements (CSTRs), which can focus on drug use, alcohol use and mental health. However, people on probation often experience negative social determinants of health and have complex health needs which are unmet by service provision (Bradley, 2009, Brooker et al., 2009, Pari et al., 2012, Pluck and Brooker, 2014, Pluck et al., 2015, Revolving Doors Agency, 2017, Sirdifield, 2012, Sirdifield et al., 2019, Sirdifield et al., 2020a, Sirdifield et al., 2020b). Health needs are often missed, and when needs are identified information about these needs and any treatment required is not consistently transferred between agencies as people progress through the CJS. There are also inconsistencies in how data are recorded, and such data rarely inform local Joint Strategic Needs Assessments and service planning (Richards, 2020). People encounter a lack of appropriate service provision and long waiting lists – something that has been exacerbated by the Covid-19 pandemic (Black, 2021, HM Inspectorate of Probation et al., 2021, HMIP and CQC, 2021, HMPPS and NPS, 2019, Sirdfield et al., 2022).

Thus, the need and rationale for supporting health improvement in the probation population is clear. However, evidence on how to do this is lacking - relatively little is known about the most effective ways of improving the health of people on probation, or about the quality of the health and social care that this population receive (Brooker et al., 2020, Sirdifield et al., 2020a, Sirdifield et al., 2020b) and there are no specific quality indicators for use in the probation service in England and Wales.

Although probation practitioners in England and Wales do not usually directly provide health or social care, they perform a health-related role. The most up-to-date description of this when the study began in 2021 was given in the NPS health and social care strategy. This states that the role includes identifying and recording health and social care needs amongst people on their caseload; supporting the development of pathways into care for people on probation including helping people to register with a GP and supporting continuity of care for those that previously received care in prison; and considering health when advising the courts on sentencing, including the use of CSTRs. Since reunification of the probation service, the role has been echoed in the *National Partnership Agreement for Health and Social Care for England* (HM Government and NHS England, 2023). Whilst broader in focus, this document also describes a role for criminal justice staff in helping people on probation to access and engage with health and social care, and acknowledges the need for reliable data on health needs to inform service delivery and measure outcomes.

In a wider study we aim to create quality indicators that are directly relevant to probation practice in terms of the health-related role described above, and provide information (for example highlighting good practice, need for improvement in service provision, or variations in outcomes) that probation practitioners could share with partner agencies as part of a quality improvement cycle.

## Methods

Ethics permission for the study was gained from the University of Lincoln (2021_6947) and the National Research Committee (2021-124).

We employed a version of the modified RAND method detailed in Van Engen-Verheul et al., (2011) as shown in Box 1. This paper presents findings from Step 3 – a review aiming to explore the breadth of existing standards, quality indicators and guidelines concerning the health and social care needs of people in the CJS, and specifically (due to the timing of the study), to identify any measures that are relevant to probation practice and the objectives outlined in the NPS health and social care strategy (HMPPS and NPS, 2019).

The probation strategy objectives are grouped into the following topics: mental health and wellbeing; substance misuse; suicide prevention; social care; physical health; learning difficulties and challenges and/or autism; the offender personality disorder pathway; and core commitments. We wished to determine the extent to which relevant measures exist in relation to the objectives within each of these topics, and the types of settings or populations they were being used with(in). Additionally, we aimed to classify the documents according to the type of information they contained: standards, indicators, guidelines, or a combination of these. Finally, we sought to identify any potential learning for the development of indicators for probation practice from papers examining the implementation of standards or quality indicators elsewhere.

### Search Strategy

We searched MEDLINE, CINAHL, Cochrane Library, IBSS, and Web of Science, with an initial search strategy being constructed on MEDLINE using a combination of keywords and subject headings (Appendix 1) and adapted for the remaining databases through an iterative process. To identify relevant papers from the grey literature, we searched the websites of the following UK-based organisations: National Institute for Health and Care Excellence (NICE), Care Quality Commission (CQC), HM Inspectorate of Probation, National Health Service (NHS), Public Health England, Department of Health and Social Care, and Clinks. Additionally, we included papers that were identified through consulting professionals in the field.

The searches were restricted to the last ten years and results (minus duplicates) were extracted into an Excel file and shared across the research team.

### Article Selection

Two researchers independently assessed papers for inclusion, with differences of opinion being resolved through discussion with a third researcher. To be included in the review, papers needed to have been published within the last ten years, and contain quality indicators, guidelines or standards for health or social care services that related to care for adult criminal justice populations (i.e., those aged 18+ years at any point in the criminal justice pathway) within one of the areas detailed in probation’s health and social care strategy. Full copies of all papers marked for inclusion were ordered.

### Data Extraction

We created a bespoke data extraction form and recorded details of the authors and year of publication; the quality indicators, guidelines or standards included in the paper; the country and part of the CJS in which they were used; and (where available) any evidence of the feasibility (barriers/enablers) for use. From this, a list of existing indicators, guidelines and standards was created and grouped by the health or social care areas within the NPS strategy.

We classified the types of measures contained within the publications using the definitions of standards, indicators and guidelines provided by Runciman et al., (2012). Flowers (2005) argues that indicators should have clear specifications - “clear and comprehensive information should be available about the construction of an indicator, including details of numerator and denominator data and the calculations necessary to derive the indicator value” (Flowers et al., 2005: 242). Documents that contained this type of ‘clearly specified’ indicator were of particular interest.

## Results

**Figure 1:**
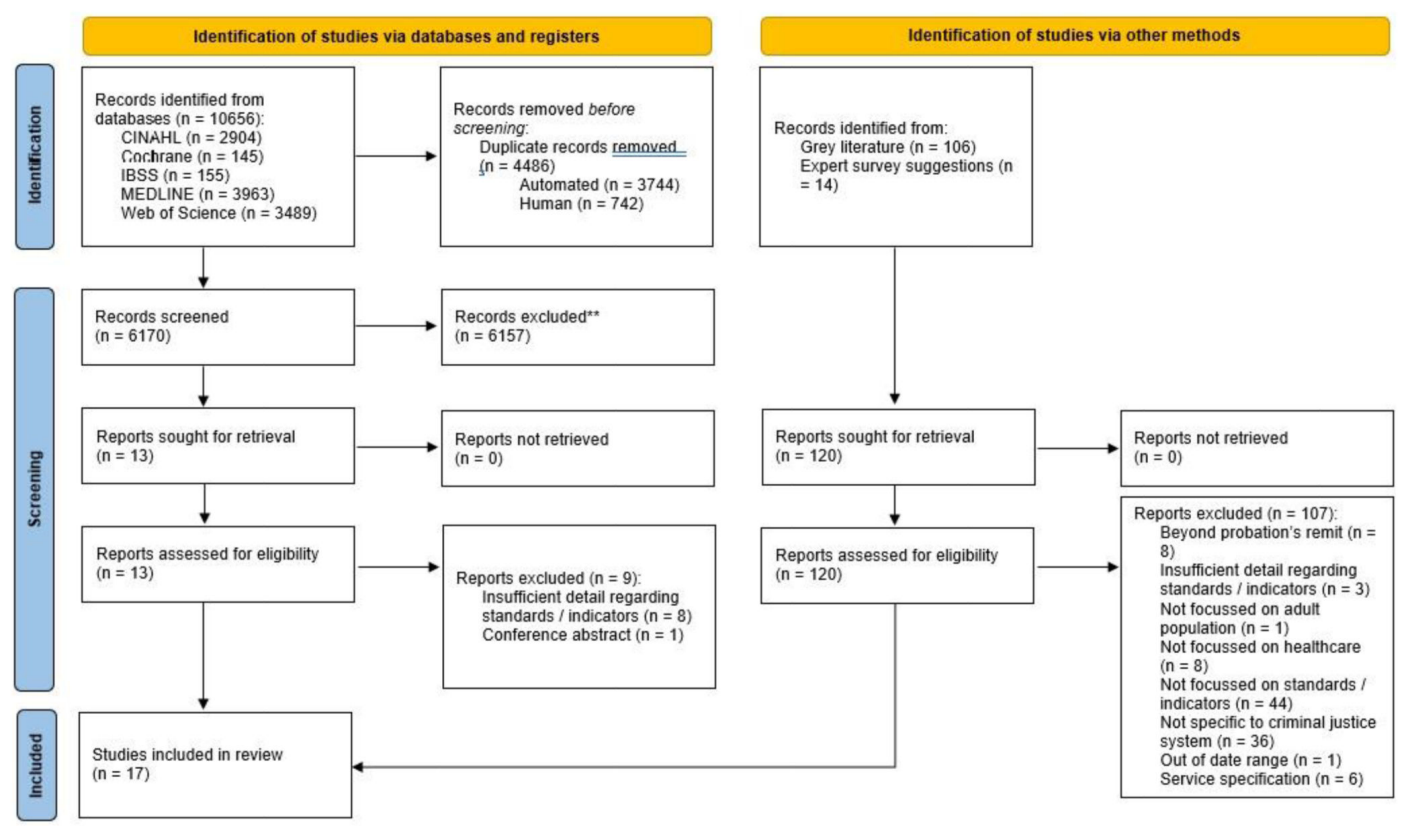
PRISMA flow diagram showing search process

The initial database search identified 10,656 papers, which reduced to 6170 once duplicates were removed. A further 120 papers were identified through other methods (106 from the grey literature search and 14 suggested by the professional panel). After screening was completed, 17 papers were identified which met the inclusion criteria (Figure 1). Of these, four were academic articles and 13 were from the grey literature. Three of the four academic papers described the process of developing standards and indicators with which to assess the quality of health care in prisons (Greifinger, 2012, McCann et al., 2020, Sawasdipanich et al., 2018). The McCann et al., (2020) paper discussed the process of developing standards to address the health of wellbeing of women in prison. This formed the basis of the PHE (2018) gender specific standards for women in prison, which is also included in this review. The remaining academic paper (Georgiou and Townsend, 2019) reviewed the level of compliance with the RCP Standards for Prison Mental Health Services (4th edition) within prisons in the UK and Northern Ireland. The papers identified from the grey literature comprised guidelines, standards and performance measures that have been published by various organisations addressing health and social care among UK criminal justice populations.

Most papers were focussed on the UK CJS, with just one paper based in the USA (Greifinger, 2012) and one focused on the Thai prison system (Sawasdipanich et al., 2018). Twelve papers focussed on health and social care within prisons, whilst just two were focussed on probation (HMIP, 2022, Scottish Government, 2016). The remaining three covered the CJS more broadly (Table 1).

Most papers (13) included standards, guidelines or indicators that we classified as being relevant to the core commitments within the NPS objectives. Numbers in relation to the remaining areas within the strategy were as follows: Mental health and well-being (11), suicide prevention (9), substance misuse (7), physical health (7). Learning disabilities and challenges and/or autism (7), social care (6), and the offender personality disorder pathway (1).

We examined how the standards, indicators or guidelines within the papers were developed. For five of the papers (HMIP, 2022, HMIP, 2017, HMIP and CQC, 2018, NHS England, 2017, NHS, 2021) the methods were unclear. Six papers^1^ either explicitly stated they had used a form of Delphi method or described similar procedures (Georgiou and Townsend, 2019, HMIP, 2021, McCann et al., 2020, Public Health England, 2018, RCP, 2021, Scottish Government, 2016). The three NICE guidelines (NICE, 2017, 2016, 2018b) were developed according to the standard procedure set out by NICE^2^ which includes literature reviews and cost analyses, and stakeholder consultation. The quality standards in NICE (2018a) were developed according to their standardised procedure^3^ which includes producing a topic overview, stakeholder engagement, and consultation on and approval of draft indicators by the Quality Standards Advisory Committee. Greifinger (2012) developed standards and indicators from existing performance measures. Beyond this, little description of the method of development is provided. Sawasdipanich et al. (2018) used a three-phase method to develop the standards of healthcare facility for Thai female inmates (SHF-TFI) - conducting interviews with female inmates and prison nurses, combining this data with information from the existing literature and feedback from public hearing meetings to develop the standards, and testing the acceptability and feasibility of the standards in practice.

We also categorised the papers as presenting or assessing either standards, indicators, guidelines, or a combination of standards and indicators, and examples of these classifications are provided for each included paper in Table 1. Five papers included standards alone. For example, the PHE (2018) gender specific standards for women in prisons presented a list of standards such as “At reception into prison, women should receive a first-stage health assessment, including physical health, alcohol use, substance misuse, mental health and self-harm and suicide risk.” This was supported by an in-depth description and rationale but was not accompanied by an indicator discussing the measurement of the standard. Two papers, Greifinger (2012) and NHS England’s (2017) health and justice indicators of performance (HJIPs), presented indicators alone, for example the HJIPs listed indicators such as “The % of patients with signs of TB infection referred to a specialist service for assessment” in isolation, without a supporting standard that the indicator was intended to measure.

Seven papers presented or assessed both standards and indicators. For example, HMIP’s (2021) Expectations paper on women in prison lists standards such as “Women’s immediate health, substance misuse and social care needs are recognised on reception and responded to promptly and effectively” followed by specific indicators, e.g., “A competent health professional screens all new women on the day they arrive to identify their immediate risks and make appropriate onward referrals” and “Immediate substance misuse needs are identified and managed, including overnight monitoring.”

Finally, three papers listed guidelines - detailed guidance for how standards should be applied in practice. For example, NICE (2017) contains guidelines for addressing mental health care of people in contact with the CJS, such as “When assessing people in contact with the criminal justice system all practitioners should: recognise potential barriers to accessing and engaging in interventions and methods to overcome these at the individual and service level; discuss mental health problems and treatment options in a way that gives rise to hope and optimism by explaining that change is possible and attainable; be aware that people may have negative expectations based on earlier experiences with mental health services, the criminal justice system, or other relevant services”.

Of the nine papers that included indicators, only five contained clearly specified methods of measurement such as the relevant numerators and denominators, or comprising dichotomous questions that would allow for some degree of quantification. An example of this is the NICE (2018a) quality standards aimed at supporting the mental health of people involved in the CJS, which include “Adults in contact with the police because of a suspected offence who have suspected mental health problems are referred for a comprehensive mental health assessment” and associated indicators, e.g., “Number of mental health assessments undertaken following referral from police services.” Here we will refer to these as ‘clearly specified indicators’.

In contrast, other papers such as the HMIP and CQC (2018) quality standards for social care in prison contain standards such as “the social care support needs of the prisoners should be met from the moment a need is identified. Prisoners should not be subject to administrative delays or unnecessarily lengthy processes.” However, the indicators used to measure these standards could be perceived as more subjective or open to interpretation e.g., “prisoners with social care needs are promptly identified and referred for a social care assessment.” Here, the indicator is a direct component of the associated standard, providing a means to identify whether the standard is being met, but the specific detail regarding how it might be measured is lacking e.g., the proportion of prisoners who are identified with a social care need within the first month.

Only one of the papers including clearly specified indicators was specific to probation. The Scottish Government (2016) framework provides standards such as “people [on probation] have better access to the services they require, including welfare, health and wellbeing, housing and employability” with the indicator “percentage of people released from a custodial sentence: registered with a GP; have suitable accommodation; have had benefits eligibility check”. This is followed up with specific information, e.g., “Should be used in conjunction with indicators around support on accessing services and interventions”, making it a clearly specified indicator.

We classified the clearly specified indicators according to what they measured. Some indicators were classified within two categories e.g., in Greifinger (2012) “Suicide screening: Do all individuals identified as needing mental health services receive a full mental health evaluation and treatment, where appropriate?” was categorised as measuring both ‘screening’ and ‘referral’. Of the 52 individual indicators, 14 were focussed on ‘service access’, 12 on both ‘service receipt’ and ‘screening’, 9 on ‘information sharing’, 7 on ‘referral’, 6 on ‘outcomes for the person on probation’, 4 on ‘collaborative working’ and one each on ‘provision of interventions’, ‘recognising needs’, ‘personalised care’, and ‘lived experience involvement’. This shows that most of the clearly specified indicators concern measuring the service availability and attendance.

Looking specifically at the one paper focussed on probation with clearly specified indicators (Scottish Government, 2016), ‘service access’ was again the most common category, with four indicators (Table 2). Additionally, two indicators measure ‘positive outcomes for the person on probation’, with one each for ‘collaborative working’, ‘personalised care’, and ‘provision of interventions’. This highlights the substantial lack of guidance for measuring health and social care in the probation population.

Just two studies investigated the feasibility of and compliance with standards. Georgiou and Townsend (2019) conducted a review of compliance with the RCP Standards for Prison Mental Health Services (fourth edition) across prisons in the UK and Northern Ireland. They found that there was a fairly stable level of compliance, with the average of around 70% the of standards being met. They highlighted an issue with mental health awareness among prison staff, finding that the percentage of services which supported training in prison decreased from 47% to 39% between the second and third years of the review. However, there was an increase in the availability of information regarding mental healthcare and related services, rising from 28% in the first year to 32% in the third. Overall, this paper demonstrates how the assessment process can be used to identify areas of good practice and areas which would benefit from targeted improvement work.

Sawasdipanich et al. (2018) developed and evaluated standards designed to improve healthcare for women in Thai prisons. The standards were piloted in one female wing of a mixed prison and one women’s prison to assess feasibility, applicability, and effectiveness. The findings showed improvements across multiple domains in both facilities following the implementation of the standards. Interviews with nurses working at the facilities suggested that they supported the standards and found them feasible to implement, however, a manual properly explaining the criteria against which the standards were scored would be helpful. A survey of the inmates revealed that most rated the healthcare services as mostly or moderately satisfactory. Whilst a focus group of inmates showed that they perceived improvements in healthcare following the implementation of the standards, though they were also able to identify further areas in need of improvement. Feasibility relied on “support from the prison warden, commitment of the staff, adequacy of medical supplies, suitability of the physical environment, and effective management” (Sawasdipanich et al., 2018: 172).

## Discussion

This review, which to our knowledge is the first of its kind, was conducted as part of a wider project to develop quality indicators that can be used as part of a plan, do, study, act cycle to support health improvement amongst people on probation and to measure key characteristics of the quality of health and social care that this population receives. The review was deliberately broad - investigating the scope of standards, indicators, and guidelines currently used to assess the quality of health and social care for adult criminal justice populations. These were classified in terms of their relevance in relation to probation objectives. The findings suggest a distinct lack of academic work in this area. Despite conducting an extensive literature search, only four academic studies in the last decade were identified which discussed the development or assessment of health and social care standards for people in criminal justice settings.

However, a strength of our approach is that we also reviewed the grey literature and sought advice from professionals working in the criminal justice and health field regarding any other relevant existing measures. This led to inclusion of an additional 13 documents, though of these, just two were specifically focused on probation (HMIP, 2022; Scottish Government, 2016). Moreover, many of the papers provided just standards or indicators alone. Only eight papers included both, whilst only five of the papers included clearly specified indicators. Setting standards is important and helps to define areas of importance or areas that require improvement. However, without also providing information on how to measure performance against that standard, it becomes a less effective tool. Overall, this suggests that there is a lack of clear guidance regarding measuring and monitoring the quality of health and social care that people on probation receive and the associated outcomes from this.

Looking at the methods used to develop the standards and indicators included in the review, the Delphi method (or similar) was most common. Additionally, NICE guidelines and quality standards are developed according to a thorough and transparent method. As such, whilst there is a lack of literature in this area, what is available has been developed according to rigorous and valid methodology which combines existing literature with the views of key stakeholders, including those with lived experience of the CJS. However, less encouraging is the fact that several papers lacked detail on methods. These papers were mainly grey literature and therefore reporting does not adhere to the structure of academic papers. One assumes that the measures are developed rigorously and thoroughly validated before being published and put into action. However, moving forwards, it may be beneficial for organisations to be more transparent in their methods to allay any concerns over validity among stakeholders that are expected to apply the proposed standards in practice.

Only five papers presented clearly specified indicators. NICE (2021) published the process they follow to develop quality standards, in which they argue that quality statements must be measurable and be accompanied by measures (indicators) that are able to be used to assess the area of care specified in the standard, including the sources of data that ought to be measured. The importance of having clearly defined methods of measurement, whether it be quantitative, qualitative, or simple dichotomous ‘yes/no’ checklists, has been made clear in the literature (Donabedian, 2005; Flowers, 2005; Mainz, 2003; NICE, 2021; Runciman, et al., 2012; Wiles, 2017). Despite this, almost half of the papers which included indicators, did not include this detail. Any future publications in this field should aim to describe the specific data that needs to be collected to objectively measure progress against each given standard.

Across the five papers which did include clearly specified indicators, there were 52 individual indicators that were relevant to the objectives set out in the NPS health and social care strategy (HMPPS and NPS, 2019) (and which remain relevant to practice within the service post-reunification). Most of these indicators related to access to health and social care services and receipt of those services. Other areas, such as screening for health and social care issues, and information sharing between departments and institutions were also covered by numerous indicators. Whilst areas such as referral into services and collaborative working were less frequently addressed. There was a relatively even spread of indicators across the NPS strategy objective areas, providing some basis for future work in developing standards and indicators to address those areas. However, only one standard was listed against the ‘learning disabilities and challenges and/or autism’ topic. Moreover, that indicator, “Existence of joint-working arrangements such as processes/protocols to ensure access to services to address underlying needs” (Scottish Government, 2016), is broad in nature. It does not specifically address the topic. We also note that that since this review was conducted, the Community Justice Performance Framework has replaced the previous Outcomes, Performance and Improvement Framework indicators from the Scottish Government, with very few indicators in the revised set directly focusing on health. The lack of probation-specific measures means it is likely that we may need to adapt the phrasing of existing standards and indicators to fit the probation context in the next stages of our research.

Only two papers examined the use of measures in practice. They point to the potential for quality assessment exercises to lead to improvements in practice and suggest that the clarity of indicators and buy-in from managerial and frontline staff is central to ensuring feasibility – something that we will consider in future work.

## Conclusion

This paper comprises one stage of a larger project aiming to develop standards and indicators to measure the quality of health and social care for people on probation. Findings suggest that there is a limited amount of existing work, especially aimed at probation, on which to base any future indicators. This is perhaps unsurprising given that probation practitioners do not usually directly provide health or social care. However, people on probation are an underserved population with a high level of health and social care needs for whom the need to improve health and access to appropriate health and social care services is clear. The government has recently published ten priorities around improving the quality of health and social care services for people supervised by probation in the community (HM Government and NHS England, 2023). We believe that addressing these priorities, the objectives within probation’s previous health and social care strategy, and the broader goal of improving the health of people on probation would be aided by the development of clear, concise standards and indicators that a) provide an objective framework, with clearly specified outcomes by which to measure improvement, and b) highlight areas where action is required. The findings from this review will be used to inform the development of such standards and indicators for probation in future stages of our research. We hope that the review also provides useful insights for individuals commissioning and providing health and social care in criminal justice settings and those with an interest in quality improvement in the CJS.

## Supplementary Material

Appendix 1

## Figures and Tables

**Table 1:** All included papers

Paper	Country	Area of CJS	Category of guidance	Example guideline, indicator, or guideline	Probation objective areas covered ^2^
Georgiou & Townsend (2019)	UK	Prison	Standards	*This paper assessed the feasibility of the ‘RCP Standards for prison mental health services - Fourth edition’ without providing example standards. The fifth edition of the standards are included in our review below*	N/A
Greifinger (2012)	USA	Prison	Indicators^1^	Do all individuals identified as needing mental health services receive a full mental health evaluation and treatment, where appropriate?	Core commitments **Suicide prevention** Physical health
McCann et al (2020) ^3^	UK	Prison	Standards	Prepare for and ensure continuity of care for women on release into the community	**Core commitments** Mental health and well-being
Sawasdipanich et al (2018) ^4^	Thailand	Prison	Standards & Indicators^1^	Standard 2.1: healthcare services provided to the female inmates, meeting the health problems and needs of the inmates. - Criterion 2.1.1: assessment of the health problems and health needs of the female inmates before providing them with health promotion services. - Description: health problems and health needs for health promotion of the female inmates should be explored before providing health promotion services. - Data/evidence: this comprised a report on the health problems and needs for the female inmates. - Scoring: with evidence=1 mark and without evidence=0 mark.	**Core commitments**
HMIP (2022)	UK	Probation	Standards & Indicators	Do the skills of staff support the arrangements and delivery of high-quality criminal justice and mental health services? - Cases are allocated to staff who are appropriately qualified and/or experienced - Staff have sufficient training to support service users with mental illness	Core commitments **Mental health and well-being**
HMIP (2021)	UK	Prison	Standards & Indicators	70. The specific needs of women with disabilities are met. - The prison accurately identifies and assess the needs of women with disabilities, including learning disabilities and other neurodiversity, and this information is kept up to date and shared appropriately with all relevant staff - Staff understand the way in which neurodiversity may present in behaviour and respond appropriately to this	Core commitments Mental health and well-being Substance misuse Suicide prevention Social care Physical health **Learning disabilities and challenges and/or autism**
HMIP (2017)	UK	Prison	Standards & Indicators	64. An effective whole-prison strategic approach to drugs and alcohol ensures the demand for drugs and alcohol is reduced - Prison officers receive training to enable them to recognise when a prisoner requires referral to substance use services, and there is a clear referral pathway - Patients with both mental and substance-related problems have prompt access to joined-up, comprehensive support	Core commitments Mental health and well-being **Substance misuse** Suicide prevention Social care Physical health Learning disabilities and challenges and/or autism
HMIP & CQC (2018)	UK	Prison	Standards & Indicators	Prisoners’ social care needs are comprehensively assessed by appropriately trained professionals - Prisoners with social care needs are identified and referred for assessment promptly (including transfer into the establishment and on the wings) - All needs are identified within the assessment - Assessments are timely, thorough and carried out in private and with the prisoner's involvement where possible	**Social care**
NICE (2017)	UK	All CJS	Guidelines	1.6.3 Providers of services should ensure staff are able to identify common features and behaviours associated with personality disorders and use these to inform the development of programmes of care.	Core commitments Mental health and well-being Suicide prevention Learning disabilities and challenges and/or autism **Offender personality disorder pathway**
NICE (2018a)	UK	All CJS	Standards & Indicators^1^	Adults with mental health problems who are in contact with the criminal justice system have a care plan that is shared with relevant services. - structure: Evidence of local arrangements for mental health care plans to include an agreed process for the plan to be shared with relevant services both inside and outside the criminal justice system - process: Proportion of adults with mental health problems in contact with the criminal justice system whose care plan is shared with the services identified in the plan as involved in their ongoing care	**Mental health and well-being**
NICE (2016)	UK	Prison	Guidelines	1.7.8 Help people who are being released from prison to find and register with a community GP if they were not previously registered with one	Core commitments Mental health and well-being **Physical health** Learning disabilities and challenges and/or autism
NICE (2018b)	UK	All CJS	Guidelines	1.9.2 After a suspected suicide in residential custodial and detention settings, undertake a serious incident review as soon as possible in partnership with the health providers. Identify how: to improve the suicide prevention action plan to help identify emerging clusters others have responded to clusters	**Suicide prevention**
NHS England (2017)	UK	Prison	Indicators^1^	Drug & Alcohol Related Treatment (DART) - Alcohol Screening; The % of patients screened for problem drinking using the AUDIT screening tool	Core commitments Mental health and well-being **Substance misuse** Suicide prevention Social care Physical health
NHS (2021)	UK	Prison	Standards	Early identification and appropriate support: People with a possible learning disability or who may be autistic need to be identified as early as possible and appropriately supported to have equal access to services and equal opportunity to progress in prison. It is recommended that healthcare staff encourage people to disclose any conditions or needs, promoting the benefits of doing so. *Outcomes* - Services can identify individuals with a possible learning disability [and/or] autism	**Learning difficulties and challenges and/or autism**
PHE (2018)	UK	Prison	Standards	7.2 Older women in prison should have an initial age-specific health and social care assessment on arrival and regular assessments thereafter.	Mental health and well-being Substance misuse Suicide prevention **Social care** Physical health
RCP (2021)	UK	Prison	Standards	19. A physical health review takes place as part of the initial assessment, or as soon as possible. Guidance: This may be completed by the physical health team, or as part of the reception process	Core commitments Mental health and well-being Substance misuse Suicide prevention **Physical health** Learning difficulties and challenges and/or autism
Scottish government (2016)	UK	Probation	Standards & Indicators^1^	Effective interventions are delivered to prevent and reduce the risk of further offending - Indicator: The delivery of interventions targeted at problem drug and alcohol use - The number of Alcohol Brief Interventions (ABIs) delivered in criminal justice healthcare settings - No of referrals from criminal justice sources to drug and alcohol specialist treatment	Core commitments **Substance misuse** Social care Physical health Learning difficulties and challenges and/or autism

Note.

1 Includes clearly specified indicators.

2 Bold indicates the objective the example was categorised as relating to.

3 The standards developed and discussed in McCann et al (2020) are those that became the PHE (2018) standards for women in prison, also included in this review.

4 The Sawasdipanich et al (2018) paper only provided one example of their standards and indicators. We categorised it based on this example, which is provided in the table.

**Table 2:** Clearly specified indicators related to probation (all from the Scottish Government, 2016)

Health and Social Care Strategy Topic and Objectives	Standards/Indicators
**Core commitments**	Outcome: People have better access to the services they require, including welfare, health and wellbeing, housing and employability ∘ Indicator: Partners have identified and are overcoming structural barriers for people accessing services. ∘ Indicator: Existence of joint-working arrangements such as processes/protocols to ensure access to services to address underlying needs*. ∘ Indicator: Initiatives to facilitate access to services. ∘ Indicator: Speed of access to mental health services. ∘ Indicator: % of people released from a custodial sentence: Registered with a GP**. Outcome: Life chances are improved through needs, including health, financial inclusion, housing and safety being addressed ∘ Indicator: Individuals have made progress against the outcome. Outcome: Effective interventions are delivered to prevent and reduce the risk of further offending ∘ Indicator: Targeted interventions have been tailored for and with an individual and had a successful impact on their risk of further offending. ∘ Indicator: The delivery of interventions targeted at problem drug and alcohol use [NHS Local Delivery Plan (LDP) Standard].
**Substance misuse**	Outcome: Effective interventions are delivered to prevent and reduce the risk of further offending ∘ Indicator: The delivery of interventions targeted at problem drug and alcohol use [NHS Local Delivery Plan (LDP) Standard].

* Also supports objectives relating to learning disabilities and challenges and/or autism, social care, substance misuse, and mental health and wellbeing.

** Also supports objectives relating to physical health.

## Box 1 Modified RAND process

- **Steps one and two:** Consulting professionals in the criminal justice and health field and individuals with lived experience of being on probation to ascertain what they think the characteristics of high-quality health and social care for people on probation are, and how they could be measured.
- **Step three:** A systematic scoping review to identify any existing quality indicators, guidelines or standards for health and social care services for adult criminal justice populations that may be transferable to the probation service context.
- **Step 4:** Combining the findings from the previous stages into a draft list of quality indicators.
- **Step 5:** Asking the professional panel to rate draft indicators and amending them based on feedback.
- **Step 6:** The professional panel discuss the amended indicators, following which any final changes are made and the panel rate the final set of indicators.

## Footnotes

1 Six papers in total, though McCann et al. (2020) and PHE (2018) discuss the same set of standards. Additionally, Georgiou & Townsend (2019) discusses an earlier version of the RCP (2021) standards.

2
https://www.nice.org.uk/process/pmg20

3
https://www.nice.org.uk/standards-and-indicators/timeline-developing-quality-standards
