# Supplementary material for: A Systematic Scoping Review of Measures of the Quality of Health and Social Care for Adults in the Criminal Justice System: Learning for the Probation Service
Source: Probat J. Author manuscript; Available in PMC 2024 Sep 20. (PMC7616584; doi:10.1177/02645505231221228)
Supplement: Appendix 1 [file EMS192586-supplement-Appendix_1.docx]

# Appendix 1: MEDLINE Search Strategy

| S1 | (MH "Prisons") OR (MH "Police") OR (MH "Criminals") OR (MH "Prisoners") |
| --- | --- |
| S2 | TI ( prison* OR probation* OR “community rehabilitation compan*” OR parole* OR police OR court* OR jail* OR “criminal justice” OR detention OR sentenc* OR remand* OR “community order*” OR “community sentenc*” OR offend* OR felon* OR convict* OR inmate* OR criminal* OR defendant* OR detain* ) OR AB ( prison* OR probation* OR “community rehabilitation compan*” OR parole* OR police OR court* OR jail* OR “criminal justice” OR detention OR sentenc* OR remand* OR “community order*” OR “community sentenc*” OR offend* OR felon* OR convict* OR inmate* OR criminal* OR defendant* OR detain* ) |
| S3 | S1 OR S2 |
| S4 | (MH "Quality of Health Care") OR (MH "Quality Control") OR (MH "Delivery of Health Care") OR (MH "Quality Indicators, Health Care") OR (MH "Quality Improvement") OR (MH "Quality Assurance, Health Care") OR (MH "Outcome and Process Assessment, Health Care") OR (MH “Process Assessment, Health Care”) OR (MH “Outcome Assessment, Health Care”) OR (MH “Standard of Care”) OR (MH “Patient Outcome Assessment”) OR (MH “Patient Reported Outcome Measures”) |
| S5 | TI ( measure* OR indicator* OR assess* OR standard* OR guideline* OR benchmark* ) AND TI ( quality OR performance OR outcome* ) OR AB ( measure* OR indicator* OR assess* OR standard* OR guideline* OR benchmark* ) AND AB ( quality OR performance OR outcome* ) |
| S6 | S4 OR S5 |
| S7 | (MH "Mental Disorders+") OR (MH "Self-Injurious Behavior") OR (MH "Suicide") OR (MH "Learning Disabilities+") OR (MH "Health+") |
| S8 | TI ( health OR “physical health” OR “mental health” OR suicide OR “social care” OR “substance misuse” OR “learning disabilit*” OR autis* OR “personality disorder*” OR healthcare N6 access* OR “health care” N6 access* OR patient N6 experience* OR patient N6 satisf* ) OR AB ( health OR “physical health” OR “mental health” OR suicide OR “social care” OR “substance misuse” OR “learning disabilit*” OR autis* OR “personality disorder*” OR healthcare N6 access* OR “health care” N6 access* OR patient N6 experience* OR patient N6 satisf* ) |
| S9 | S7 OR S8 |
| S10 | S3 AND S6 AND S9 |
| S11 | Limiters: last 10 years |
